# Dietary Strawberries Improve Serum Metabolites of Cardiometabolic Risks in Adults with Features of the Metabolic Syndrome in a Randomized Controlled Crossover Trial

**DOI:** 10.3390/ijms24032051

**Published:** 2023-01-20

**Authors:** Arpita Basu, Kenneth Izuora, Andrew Hooyman, Hal R. Scofield, Jeffrey L. Ebersole

**Affiliations:** 1 Department of Kinesiology and Nutrition Sciences, University of Nevada at Las Vegas, Las Vegas, NV 89154, USA; 2 Section of Endocrinology, University of Nevada School of Medicine, Las Vegas, NV 89154, USA; 3 School of Biological Health Systems Engineering, Arizona State University, Tempe, AZ 85281, USA; 4 Section of Endocrinology and Diabetes, University of Oklahoma Health Sciences Center, Oklahoma City, OK 73104, USA; 5 Research and Medical Services, Department of Veterans Affairs Medical Center, Oklahoma City, OK 73104, USA; 6 Arthritis and Clinical Immunology, Oklahoma Medical Research Foundation, Oklahoma City, OK 73104, USA; 7 School of Dental Medicine, University of Nevada at Las Vegas, Las Vegas, NV 89154, USA

**Keywords:** strawberries, obesity, branched-chain amino acids, serum phosphate, insulin resistance, serum hydroxyphenyl propionic acid

## Abstract

Dietary strawberries have been shown to improve cardiometabolic risks in multiple clinical trials. However, no studies have reported effects on serum metabolomic profiles that may identify the target pathways affected by strawberries as underlying mechanisms. We conducted a 14-week randomized, controlled crossover study in which participants with features of metabolic syndrome were assigned to one of the three arms for four weeks separated by a one-week washout period: control powder, 1 serving (low dose: 13 g strawberry powder/day), or 2.5 servings (high dose: 32 g strawberry powder/day). Blood samples, anthropometric measures, blood pressure, and dietary and physical activity data were collected at baseline and at the end of each four-week phase of intervention. Serum samples were analyzed for primary metabolites and complex lipids using different mass spectrometry methods. Mixed-model ANOVA was used to examine differences in the targeted metabolites between treatment phases, and LASSO logistic regression was used to examine differences in the untargeted metabolites at end of the strawberry intervention vs. the baseline. The findings revealed significant differences in the serum branched-chain amino acids valine and leucine following strawberry intervention (high dose) compared with the low-dose and control phases. Untargeted metabolomic profiles revealed several metabolites, including serum phosphate, benzoic acid, and hydroxyphenyl propionic acid, that represented improved energy-metabolism pathways, compliance measures, and microbial metabolism of strawberry polyphenols, respectively. Thus, dietary supplementation of strawberries significantly improves the serum metabolic profiles of cardiometabolic risks in adults.

## 1. Introduction

Metabolic syndrome, defined as a cluster of cardiometabolic risk conditions, includes a large waist circumference, dyslipidemia, elevated blood pressure, as well as insulin resistance and impaired glucose tolerance, and has been identified as a major risk factor for type 2 diabetes and its cardiovascular complications [1]. Thus, several dietary strategies, especially the use of different forms of dietary supplements, continue to be investigated for the prevention and management of this condition. Among the dietary supplements, plant-derived bioactive compounds or phytochemicals have been shown to improve multiple features of metabolic syndrome in experimental and human studies [2,3]. Certainly, the phytochemicals in fruits and vegetables account for most of their health benefits in reducing metabolic risks [4,5]. Among the dietary fruits, berries, such as cranberries, blueberries, and strawberries, have been reported to improve the features of metabolic syndrome in many clinical studies [6]. These findings are especially important in the context of the poor dietary quality of US adults [7,8] and the need to identify the role of single foods or dietary supplements that are feasible in improving metabolic outcomes without changing the entire diet.

The human serum metabolome represents a plethora of compounds that are largely affected by diet and lifestyle factors, as well as the gut microbiome [9,10,11,12]. Targeted profiling also reflects causality and thus indicates how dietary compounds may affect specific nutrient metabolism pathways, thereby guiding effective recommendations. Rodent models of obesity, as well as prediabetes or metabolic syndrome, have revealed significant changes in ketone bodies and mitochondrial bioenergetic metabolites following supplementation with blueberry juice [13] and increases in polyphenol metabolites following supplementation with freeze-dried strawberries [14]. However, few clinical reports exist on the role of dietary phytochemical/polyphenol or berry interventions on the serum profiles of carbohydrate and lipid metabolites in adults with obesity and metabolic syndrome. Following an eight-week polyphenol-rich-diet intervention in older healthy adults, there were significant increases in serum polyphenol metabolites and a decrease in specific metabolites associated with lipid metabolism when compared with the control diet [15]. In another study of bilberry supplementation in adults with metabolic syndrome, hippuric acid increased significantly as a serum metabolite of berry consumption vs. the control group [16]. A systematic review of clinical trials involving blueberries and strawberries revealed human serum metabolomic signatures that were indicative of the specific berry intervention: 2-hydroxybenzoic acid and hippuric acid for the blueberry intervention and 3-methoxyphenylacetic acid and 4-hydroxyphenylacetic acid for the strawberry intervention [17]. However, these studies did not examine the effects of berry intervention on the metabolites of macronutrient pathways, which are of great relevance in understanding the molecular mechanisms of dietary berry intervention in cardiometabolic conditions.

In our previously reported studies, strawberry supplementation was shown to decrease the total and LDL cholesterol in overall healthy adults with abdominal obesity and dyslipidemia [18,19]. In our more recent randomized crossover trial in adults with features of metabolic syndrome, dietary strawberry supplementation (~ 2.5 servings daily for four weeks) significantly decreased insulin resistance when compared with the control phase [20]. Thus, using serum samples from the same trial, we now aim to examine the serum metabolites (targeted and untargeted) that may be affected by strawberry supplementation. We hypothesized that dietary strawberry supplementation, administered as freeze-dried strawberry powder, would affect the pathways of glycemic control and insulin secretion that may explain the previously observed clinical outcome of improved insulin resistance in adults with features of metabolic syndrome.

## 2. Results

### 2.1. Baseline Characteristics and Compliance

Table 1 presents the baseline characteristics of the 33 participants who completed all three phases of the randomized crossover study. Protocol compliance was 96%. Plasma ellagic acid, as a measure of compliance, revealed the following levels (means ± SD): baseline: non-detectable; control: non-detectable; low-dose strawberry: 15±8nmol/L; high-dose strawberry: 27 ± 13 nmol/L.

### 2.2. Features of Metabolic Syndrome

The features of metabolic syndrome, including body weight, waist circumference, blood pressure, blood glucose, triglycerides, and HDL-cholesterol levels, did not differ at the end of each treatment group, as previously reported [20]. Analysis by strawberry treatment revealed significant improvements in fasting insulin and insulin resistance in the high-dose strawberry (mean ± SD: 9.1 ± 3.1 and 2.1 ± 0.5, respectively) vs. low-dose strawberry (14.0 ± 8.2 and 3.3 ± 2.0, respectively), control (15.2 ± 6.4 and 3.5 ± 1.4, respectively), and baseline (15.4 ± 6.6 and 3.6 ± 1.5, respectively) phases (all *p* < 0.05). 

### 2.3. Serum Targeted Metabolites of Primary and Lipid Metabolism Pathways

Table 2 shows the serum levels of the targeted metabolites after each phase of the randomized, controlled crossover trial. Among the targeted metabolites, the serum levels of the branched-chain amino acids (BCAAs) valine and leucine were significantly lower following the high-dose strawberry phase when compared with the control and low-dose strawberry phases (*p* < 0.05). No significant differences were noted in the cases of serum isoleucine, fatty acids, citric acid, and alanine when examined by treatment phases.

### 2.4. Habitual Dietary Intakes and Flavonoid Intakes

Table 3 shows the background dietary intakes of total calories and macronutrients, which were not significantly different between the different phases of the crossover study. Furthermore, analyses of the major categories of dietary flavonoid intakes based on the USDA flavonoid database also revealed no significant differences arising from habitual dietary intakes. 

### 2.5. Untargeted Metabolites

**Figure 1:**
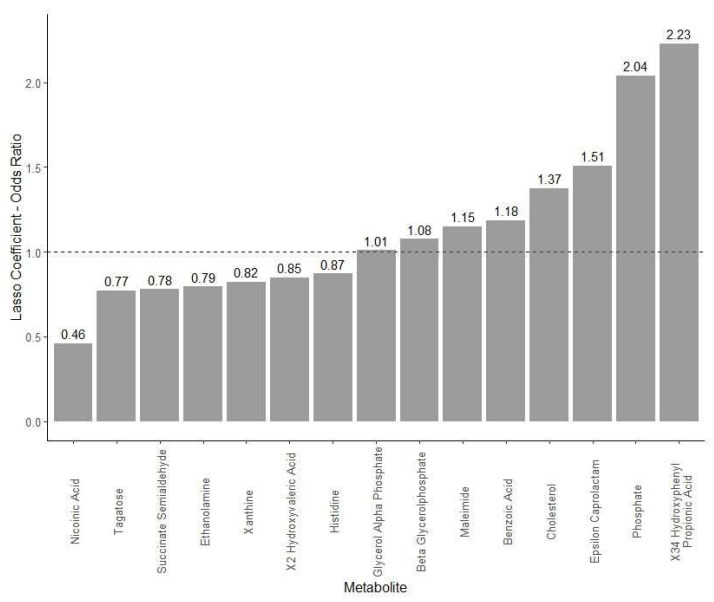
LASSO logistic regression analyses comparing significant metabolites (untargeted platform) at baseline and after strawberry intervention in adults with features of metabolic syndrome.

Figure 1 shows the 15 significant metabolites that were predictive of strawberry intervention when compared with the baseline. Among these 15, greater values of serum glycerol alpha phosphate, beta glycerophosphate, maleimide, benzoic acid, cholesterol, epsilon caprolactam, phosphate, and hydroxyphenyl propionic acid were significantly associated with the classification of the strawberry intervention. On the other hand, greater levels of serum nicotinic acid, tagatose, succinate semialdehyde, ethanolamine, xanthine, hydroxyvaleric acid, and histidine were significantly associated with classification to the baseline levels.

## 3. Discussion

In our clinical trial of dietary strawberry supplementation in adults with features of metabolic syndrome and insulin resistance, several targeted and untargeted serum metabolites were shown to be modulated. When analyzed by treatment phases, the serum valine and leucine were significantly lower following the high-dose (2.5 servings/day) strawberry phase when compared with the low-dose and control phases. Untargeted metabolites revealed a significant group of compounds that were predictive of strawberry intake when compared with the baseline; elevated levels of hydroxyphenyl propionic acid indicated colonic degradation of strawberry phytochemicals, as well as an increase in propionic acid that has been associated with improved insulin resistance and metabolism. Elevated serum phosphate, as a primary metabolite, indicates the role of strawberry supplementation in improving energy-related pathways in tissue metabolism.

Branched-chain amino acids (BCAAs) have been correlated with energy and glucose metabolism, and the circulating levels of leucine, isoleucine, and valine have been shown to be elevated in obesity and predict insulin resistance and diabetes [21,22]. Thus, medications in diabetes management have been shown to lower circulating BCAAs [23]. To our knowledge, this is the first clinical study to identify the role of dietary polyphenol-rich strawberries in modulating serum BCAAs. In general, few clinical data have been reported on the role of plant-based diets, food groups, or phytochemicals in modulating BCAAs. In a group of healthy omnivorous adults, the intake of a vegan diet for four weeks significantly decreased BCAAs when compared with a meat-based diet [24]. Isoleucine, leucine, and valine were significantly lower after four weeks following a vegan vs. a meat-based diet in these adults with healthy body weight. A Mediterranean diet, characterized by high consumption of plant-based foods and limited consumption of red meat and processed foods, has also shown similar results in clinical trials. In the PREDIMED trial, supplementation of the Mediterranean diet with extra virgin olive oil or nuts reduced the incidence of type 2 diabetes, as well as plasma BCAAs, when compared with the control group; the baseline BCAAs predicted diabetes incidence in the control, but not in the diet group in adults at risk [25]. Data from animal models of diet-induced obesity or diabetes also revealed that polyphenol-rich foods and beverages, such as coffee, resveratrol, and curcumin, resulted in decreasing circulating BCAAs when compared with the control groups [26,27]. While clinical data are limited in this area, our observations on the role of dietary strawberries in lowering serum BCAAs corroborate these previous findings and must be investigated in larger trials.

To our knowledge, no previous study has reported the effects of strawberry or other berry supplementation on serum metabolomics associated with the energy metabolism and lipid metabolism pathways in adults with features of metabolic syndrome. Our findings show that whole strawberries can lower selected BCAAs that can improve insulin resistance and the energy/lipid metabolism pathways, and lower diabetes risk [28,29]. Our data add to the emerging evidence on the role of fruit polyphenols, especially anthocyanins in strawberries, in improving diabetes risk via different mechanisms that are usually observed in animal models and have now been confirmed in our clinical study [5,30,31]. This has important implications, as we used whole strawberries and not isolated polyphenol supplements, which emphasizes the role of whole fruits rich in a variety of polyphenols and nutrients in a healthy diet.

Among the untargeted metabolites, several compounds were shown to be modulated following the strawberry-supplementation phase. The serum levels of phosphate-containing metabolites, such as glycerol phosphate and phosphate, as well as cholesterol, were significantly predictive of strawberry supplementation, thereby showing the role of strawberry polyphenols in upregulating glycolysis-related lipids and energy metabolism pathways. While serum LDL-cholesterol has been shown to be lowered by berry and/or polyphenol supplementation in adults in previous studies [18,32], our findings of an increase in serum cholesterol as a metabolite may indicate altered hepatic cholesterol synthesis, perhaps via altered bile acid synthesis/reabsorption, as observed in mechanistic studies [33,34]. These metabolomics data further reveal differences in the cholesterol profiles in adults with different cardiometabolic profiles, such as general metabolic syndrome vs. elevated serum LDL-cholesterol and their responses to dietary whole-berry supplementation. Furthermore, we did not examine liver health status in our study, and underlying non-alcoholic fatty liver, quite common in adults with obesity and metabolic syndrome, may modify the circulating cholesterol levels, as observed in preclinical models [35]. The serum phosphate level was significantly higher following strawberry intervention as observed in our study. Lower serum phosphate has been associated with obesity and insulin resistance in observational studies [36,37,38,39]. Consequently, the higher serum phosphate following strawberry intervention when compared with the baseline suggests improvements in insulin resistance, as observed previously [20], and improved energy generation pathways in these adults with obesity and features of metabolic syndrome. Reported data, though limited, show the role of strawberries in improving insulin resistance following a high-fat meal [40], and our findings shed some light on its underlying mechanisms, which are possibly related to the increasing mobilization of serum phosphate and its availability for adenosine triphosphate generation. We also observed significantly higher levels of benzoic acid and hydroxyphenyl propionic acid following strawberry intervention when compared with the baseline. Strawberries are a rich source of phenolic acids, including benzoic acid, and this was certainly a marker of strawberry intervention and compliance in our study [17]. Hydroxyphenyl propionic acid is a microbial metabolite of polyphenols, such as quercetin present in strawberries [41], and various studies have shown its role in preventing endothelial dysfunction and hypertension in experimental models [42,43,44]. Thus, this metabolite may be a major link between strawberry consumption and improvements in cardiometabolic risks, as observed in clinical trials [6,45].

Overall, our study provides unique mechanistic data underlying the effects of strawberry consumption on serum metabolic profiles that have not been previously reported. We also examined differences in habitual macronutrient and flavonoid intakes after each study phase, and in the absence of any significant differences in habitual diet, the observed differences could be attributed to strawberry supplementation. Some limitations of our study include the inclusion of adults with obesity and features of metabolic syndrome and those not taking medications for lowering glucose, lipids, and/or blood pressure, which reduces generalizability to adults on multiple medications and disease risks. We did not analyze serum metabolites according to the features of metabolic syndrome in each patient, which would require a larger sample size and thus must be examined in future studies. Additionally, we did not measure gut microbiome profiles separately in this study; thus, the serum profiles may not distinguish between metabolites derived from strawberries vs. those derived from the microbial metabolism of these compounds. Thus, future studies in adults with metabolic syndrome must examine these differences to determine how strawberries may modulate pathogenic metabolites contributing to this condition. Finally, our study cohort was predominately women; thus, these data may not be applicable to men. However, excluding the two male participants from the analysis did not affect the significant study findings.

## 4. Materials and Methods

This was a randomized, double-blind, controlled crossover trial conducted at the Oklahoma Clinical and Translational Sciences Institute (OCTSI) at the University of Oklahoma Health Sciences Center (OUHSC), Department of Nutritional Sciences at Oklahoma State University (OSU), and the Section of Endocrinology at the University of Nevada at Las Vegas (UNLV) School of Medicine, as previously described [20]. The clinical trial was approved by the ethics committees at OUHSC and UNLV and was registered at Clinicaltrials.gov (Identifier: NCT03441620). All participants provided written informed consent.

### 4.1. Study Criteria and Protocol

The study design has been previously reported [20]. Briefly, adult participants with one or more features of metabolic syndrome [1], abdominal adiposity (waist circumference: men >40 inches; women>35 inches), body mass index (BMI) in the obese range (≥30 kg/m^2^), and elevated serum LDL-cholesterol (LDL-C) > 116 mg/dL were enrolled in the study. The exclusion criteria were the current use of medications that may influence glucose and lipid metabolism (metformin, statins, glucocorticoids, immunosuppressants, and antipsychotics), unwillingness or inability to provide written informed consent, a significant underlying medical disorder assessed by the study physician (e.g., anemia, renal disorders, and diabetes), allergy to strawberries, and smokers. In the randomized crossover design, each participant completed three phases of four weeks each of the consumption of a control powder or freeze-dried strawberry powders equivalent to one serving or two and a half servings of strawberries each day. There was a one-week washout phase between each phase. The participants were instructed to follow their usual diet and lifestyle habits throughout the 14-week study and submitted dietary recalls which were then assessed for habitual nutrient intake using the ESHA’s Food Processor^®^ Nutrition Analysis software (version 7.2); the habitual flavonoid intake was assessed using the USDA flavonoid database [46].

### 4.2. Intervention and Control Powders

The compositions of the control and freeze-dried strawberry powders, provided by the California Strawberry Commission (Watsonville, CA, USA), were as follows: the low-dose strawberry group (one serving/day) received approximately 123 kcal, 28 g carbohydrates, 2 g dietary fiber, 26 mg vitamin C, 400 mg total polyphenols, and 38 mg total anthocyanins per day for four weeks, and the high-dose strawberry group (two-and-half servings/day) received approximately 124 kcal, 27 g carbohydrates, 5 g dietary fiber, 65 mg vitamin C, 960 mg total polyphenols, and 92 mg total anthocyanins per day for four weeks. The control powder was formulated to match the sensory and caloric properties of the strawberry powder, but had no polyphenol content [20].

### 4.3. Biochemical Analyses

The serum levels of glucose, insulin, and lipids were analyzed at the University of Oklahoma Medical Center laboratory (Oklahoma City, OK, USA) and at Quest Diagnostics (Las Vegas, NV, USA) according to the manufacturer’s protocols. Serum glycated hemoglobin was analyzed with the use of a DCA 2000+ Analyzer (Bayer, Leverkusen, Germany). Insulin resistance was evaluated by HOMA-IR and was calculated as follows: [fasting insulin (mU/L) × fasting glucose (mmol/L)]/22.5 [47]. In addition, sera were stored at −80 °C for the subsequent analyses of metabolic profiling. Plasma ellagic, acid as a measure of compliance, was measured using a previously published procedure [48]. Briefly, 500 µL of plasma was treated with acetonitrile and the resulting supernatant was evaporated, reconstituted with methanol, and injected into an HPLC system. Ellagic acid was eluted using a C18 column and quantified using the Millennium Chromatography software (Waters, Milford, MA, USA).

### 4.4. Serum Metabolomic Assay

Serum samples were sent to the West Coast Metabolomics Center (Davis, CA, USA) for the analyses of primary metabolites and complex lipids by combining three analytical platforms, including gas chromatography–time-of-flight mass spectrometry (Ann Arbor, MI, USA), hydrophilic interaction chromatography–quadrupole time-of-flight tandem mass spectrometry (Davis, CA, USA), and charged-surface hybrid–quadrupole time-of-flight tandem mass spectrometry (Davis, CA, USA). Internal standards were employed for the identification of metabolites by matching the retention times as previously described [49,50]. Briefly, 65 µL of a plasma sample was treated with acetonitrile and deproteinized, and the supernatant was loaded into autosampler vials. Mass spectral data were collected with a 10 min gradient from a mass/charge ratio of 85 to 2000 in the positive ionization mode. Three replicates were run for each sample using dual-column chromatography with C18 and an anion exchange column. Data quantification was performed using the adaptive processing software (apLCMS) [51]. Our current analyses focused on 9 targeted metabolites and 151 metabolites identified in the untargeted analysis of the serum samples following strawberry intervention.

### 4.5. Statistical Analyses

The summary statistics are presented as means ± SD for continuous variables and counts and percentages for discrete variables. Our primary objective was to examine if the targeted metabolites were different after each of the strawberry vs. control phases as well as the baseline. To assess differences, we used a mixed-model ANOVA to examine the main effects of the treatment, time, and interaction to examine the differences in outcomes at the end of each four-week phase of intervention. Baseline values were included as covariates for each outcome variable. The outcomes were modeled as repeated measures, with the subject as a random effect and with unstructured variance for treatment/time. The sequence of intervention was included in all models to test for carry-over effects, and none were detected. We also examined differences in habitual dietary nutrients and flavonoids using the same mixed-model approach. To examine untargeted metabolites, the LASSO logistic regression model was used to identify significant metabolites that were predictive of the strawberry treatment phases compared with the baseline. The LASSO model was trained on 80% of the total dataset and then tested on the remaining 20%. A receiver operating characteristic curve analysis was performed on the metabolites related to strawberry intervention individually in the test set data. The data were auto-scaled, and the odds ratios are presented for each significant metabolite for comparisons between the baseline and strawberry-treatment phases. The power for this study was calculated based on previous differences in serum LDL-cholesterol to achieve 80% power at 0.05 α level [18,19]. All *p*-values were 2-tailed, and main effects and interaction effects were considered if <0.05. Analyses were performed using SAS (version 9.4; SAS Institute Inc., Cary, NC, USA) and R (version 4.0.3, R Core Team, Vienna, Austria).

## 5. Conclusions

Our clinical study supports the role of strawberry supplementation in improving the serum metabolic profiles associated with decreased risks of insulin resistance and diabetes, as well as endothelial dysfunction in adults with features of metabolic syndrome. The significant decreases in the targeted metabolites, such as serum BCAAs, as well as significant increases in untargeted metabolites, including serum phosphate, benzoic acid, and hydroxyphenyl propionic acid, represent underlying pathways involved in reducing the risks of cardiometabolic dysfunction. Thus, adding whole strawberries to the habitual diet may be a beneficial and feasible strategy to improve the cardiometabolic health of adults.

## Figures and Tables

**Table 1:** Baseline characteristics of the study participants in the clinical trial.

N	33
Age (y)	53 ± 13
Sex (M/F)	2/31
BMI (kg/m^2^)	33 ± 3
Body weight (kg)	86 ± 10.5
Waist circumference (m)	1.02 ± 0.07
Serum HbA1c (%)	5.5 ± 0.3
Serum fasting glucose (mg/dL)	93 ± 13
Serum insulin (µIU/mL)	15.4 ± 6.6
Serum HOMA-IR	3.6 ± 1.5
Serum LDL-cholesterol (mg/dL)	144 ± 25
Serum triglycerides (mg/dL)	124 ± 66
Serum HDL-cholesterol (mg/dL)	54 ± 10
Blood pressure medication use, n (%)	6 (18)
Antidepressant use, n (%)	8 (24)
Multivitamin use, n (%)	5 (15)
Meeting exercise recommendations (%) ^1^	11 (33)

Data are presented as means and standard deviations (SD). Count data presented as n (%); M = male; F = female; BMI = body mass index; ^1^ ≥150 min of moderate and/or ≥90 min vigorous exercise/week.

**Table 2:** Targeted serum metabolite profiles in obese adults with above-optimal serum LDL-cholesterol following each treatment period in a randomized crossover study.

Variable	Baseline	Control (4-Week)	Strawberry (LD) (4-Week)	Strawberry (HD) (4-Week)	^1^*p*-Value (Treatment)
Valine, ng/µL	27.9±16.8 ^a^	31.4±22.8 ^a^	23.1±15.5 ^a^	17.4±11.5 ^b^	0.03
Leucine, ng/µL	29.6±13.6 ^a^	35.8±17.5 ^a^	27.4±18.4 ^a^	20.3±17.4 ^b^	0.01
Isoleucine, ng/µL	33.7±24.7	29.5±14.7	28.3±15.7	27.2±17.8	0.23
Alanine, ng/µL	66.9±25.6	78.3±19.5	71.8±23.6	75.2±33.6	0.32
Citric acid, ng/µL	12.5±7.8	10.8±6.8	9.3±7.8	13.6±9.5	0.18
Oleic acid, ng/µL	35.8±14.7	45.1±21.6	38.5±15.7	36.2±14.3	0.21
Linolenic acid, ng/µL	52.7±13.4	45.3±9.7	48.2±11.4	49.2±8.4	0.34
Linoleic acid, ng/µL	53.8±23.6	56.2±17.3	45.2±24.6	47.3±25.7	0.33
Docosahexaenoic acid, ng/µL	17.5±8.4	12.3±11.4	15.8±12.4	14.6±13.5	0.21

Data presented as means ± SD. N = 33/group; HD = high dose (~ 2.5 servings of strawberries/day); LD = low dose (~1.0 serving of strawberries/day); ^1^*p* for main effect of treatment from MIXED procedure (SAS version 9.4; SAS Institute Inc., Cary, NC, USA) adjusted for baseline values. Different superscript letters show significant differences between treatment groups for each variable; *p* < 0.05 in bold font.

**Table 3:** Dietary habitual macronutrient and flavonoid intake following each treatment period in a randomized crossover study.

Variable	Baseline	Control (4-Week)	Strawberry (LD) (4-Week)	Strawberry (HD) (4-Week)	^1^*p*-Value (Treatment)
Calories, kcal	2012 ± 152	1988 ± 183	2123 ± 113	2067 ± 193	0.74
Carbohydrates, g	226 ± 13.0	238 ± 15.4	234 ± 21.7	238 ± 18.6	0.54
Total fats, g	76 ± 13	80 ± 17	83 ± 21	85 ± 25	0.42
Proteins, g	101 ± 35	85 ± 41	106 ± 45	88 ± 39	0.28
Total flavonoids, mg	75 ± 55	82 ± 47	86 ± 38	91 ± 65	0.34
Flavan-3-ols, mg	12 ± 8	15 ± 10	9 ± 8	11 ± 10	0.32
Anthocyanins, mg	25 ± 15	20 ± 13	28 ± 16	31 ± 20	0.42
Flavanones, mg	17 ± 11	23 ± 14	21 ± 10	22 ± 14	0.36

Data presented as means ± SD. N = 33/group; HD = high dose (~2.5 servings of strawberries/day); LD = low dose (~ 1.0 serving of strawberries/day); ^1^*p* for main effect of treatment from MIXED procedure (SAS version 9.4; SAS Institute Inc., Cary, NC, USA) adjusted for baseline values.

## Data Availability

These data are not publicly available as the funding agency requires only the authorized research team to collect and store data.
